# Neurofilament levels in patients with neurological diseases: A comparison of neurofilament light and heavy chain levels

**DOI:** 10.1002/jcla.22948

**Published:** 2019-06-14

**Authors:** Pavlína Kušnierová, David Zeman, Pavel Hradílek, Martin Čábal, Olga Zapletalová

**Affiliations:** ^1^ Department of Clinical Biochemistry, Institute of Laboratory Diagnostics University Hospital Ostrava Ostrava Czech Republic; ^2^ Clinic of Neurology University Hospital Ostrava Ostrava Czech Republic

**Keywords:** axonal damage, cerebrospinal fluid, enzyme‐linked immunosorbent assay, neurofilament, neurological disease

## Abstract

**Background:**

Neurofilaments are the major cytoskeletal components of neurons, and cell injury leads to their release into the surrounding area. The aim of this study was to compare the cerebrospinal fluid (CSF) and serum (S) concentrations of neurofilament light chains (NFLs) and phosphorylated neurofilament heavy chains (pNFHs).

**Methods:**

Neurofilament concentrations were measured in CSF and S samples from 172 patients using three enzyme‐linked immunosorbent assays. Excel, Stata version 13, MedCal version 17.9.7., and NCSS 2007 software were used for the statistical analysis.

**Results:**

There was a statistically significant correlation between the concentrations of CSF NFL and CSF pNFH (*r*_s_ = 0.748; n = 89; *P* < 0.001), but Passing‐Bablok regression showed systematic deviation between the values obtained using the two assays. This indicates that the assays were not interchangeable. CSF pNFH and S pNFH concentrations showed low correlation. The kappa statistic showed moderate conformity between CSF pNFH and CSF NFL concentrations (*κ* = 0.556).

**Conclusions:**

The CSF NFL and CSF pNFH assays gave clinically consistent results that reflected the degree of axonal damage, independent of any particular neurological diagnosis. The S pNFH assays had a lower predictive value due to the low correlation coefficient and the kappa index of the CSF pNFH method.

## 1 INTRODUCTION

Neurofilaments (NFs) are the main structural proteins of neurons and are members of the class IV intermediate filament protein family. NFs are selectively expressed in the nervous system and are found at the highest levels in long projection axons. They are composed of four subunits, namely NF light (NFL), NF medium (NFM), and NF heavy (NFH) chain subunits plus an unstable alpha‐internexin subunit. These subunits have different molecular weights and functional properties. The NFL gene is on chromosome 8p21, and the NFL protein, which has a molecular mass of 61.5 kDa, consists of 543 amino acids. The NFM gene is also on chromosome 8p21; it consists of 916 amino acids and is important for radial axonal growth. The NFH gene is on chromosome 22q12.2, and the protein, which has a molecular mass of 112.5 kDa, consists of 1020 amino acids. NFH is important for protein‐protein interactions, which are regulated locally in the axon by phosphorylation.^1^, ^2^ The alpha‐internexin protein has a molecular mass of 66 kDa and can form homopolymers; however, due to its instability, this subunit is difficult to detect in laboratory practice. Its gene is on chromosome 10q24.33.

Enzyme‐linked immunosorbent assays (ELISAs) or more sensitive techniques, such as electrochemiluminescence immunoassays and single molecule arrays (SIMOAs), can be used to determine NF levels.^3^ After axonal injury, NFs are released into the extracellular space. Accordingly, their concentration in CSF and/or S reflects the degree of axonal damage.^4^ The levels of both NFL and NFH are increased in multiple sclerosis (MS), reflecting both neuroaxonal damage in active plaques, which is mediated by the inflammation, and neurodegeneration.^5^ In patients with clinically isolated syndrome (CIS), the NFL levels correlate with radiological signs of disease activity (gadolinium‐enhancing magnetic resonance lesions) and predict conversion to clinically definite MS with a worse prognosis.^6^, ^7^, ^8^ During MS progression, NFH levels correlate with physical disability and changes in brain volume but not with lesion number or volume. The NFH concentration may indicate ongoing neurodegeneration.^5^, ^7^, ^9^

Natalizumab‐treated patients show a 3‐fold decrease in NFL, indicating that this treatment not only has an immunomodulatory effect but may also reduce axonal damage.^10^ These effects are also observed in patients with MS who were treated with rituximab, mitoxantrone, or fingolimod.^11^, ^12^ However, studies have not demonstrated conclusively that the decline in axonal involvement is not secondary, and anti‐NF antibody levels do not correlate with the clinical variants of MS.^13^, ^14^

The aims of the study were to compare the cerebrospinal fluid (CSF) concentrations of NFL and pNFH and the CSF and S concentrations of pNFH and to evaluate the correlation of these parameters with the following diagnoses: MS; CIS; inflammatory diseases of the peripheral nervous system (IDPNS); and other inflammatory central nervous system diseases (OIND), noninflammatory neurological diseases (NIND), and no evidence of organic nervous system disease (the control group, Control).

## 2 MATERIALS AND METHODS

### 2.1 Patients

We examined a total of 172 patients from the Moravian‐Silesian region of the Czech Republic who had CSF and S samples sent for analysis to the Institute of Laboratory Diagnostics, Department of Clinical Biochemistry, University Hospital Ostrava. The study was approved by the Ethics Committee of the University Hospital Ostrava, Czech Republic, and was conducted in accordance with the ethical standards of the Helsinki Declaration of 1975 as revised in 2000. The average age of the entire group of subjects was 47.0 ± 16.44 years. The group consisted of 113 women (65.7%) with an average age of 46.4 ± 16.30 years and 59 men (34.3%) with an average age of 48.2 ± 16.64 years. The files of patients from the University Hospital Ostrava with available clinical data (n = 101) were further subdivided into diagnosis groups: MS (n = 19; 14 women, average age 38 ± 9.36 years; 5 men, average age 31 ± 7.30 years), CIS (n = 11; 9 women, average age 34 ± 11.12 years; 2 men, average age 31 ± 3.50 years), OIND (n = 10; 4 women, average age 39 ± 11.88 years; 6 men, average age 63 ± 6.10 years), IDPNS (n = 5; 5 men, average age 51 ± 13.06 years), NIND (n = 38; 25 women, average age 54 ± 15.87 years; 13 men, average age 58 ± 10.58 years), and Control (n = 33; 24 women, average age 43 ± 16.04 years; 9 men, average age 43 ± 16.84 years). For diagnosis of multiple sclerosis, we used the 2017 Revisions of the McDonald Criteria.^15^ The diagnoses in the OIND group comprised neuromyelitis optica (n = 3), encephalitis (n = 1), granulomatosis with polyangiitis (n = 2), aseptic meningitis (n = 1), neuroborreliosis (n = 3). The NIND group included a very wide and heterogeneous spectrum of diagnoses; more frequent were neurodegenerative diseases (n = 14), noninflammatory polyneuropathy (n = 8), and vascular CNS disease (n = 5), further CNS tumors (n = 2), vertigo (n = 3), tinnitus (n = 1), radiculopathy (n = 2), anisocoria (n = 1), spinal stenosis (n = 1), spondylogenic cervical myelopathy (n = 1). All subjects provided written informed consent for the use of their biological material (CSF and S) for research purposes. Apart from sex and age, all patient data were anonymous.

### 2.2 Samples

Neurofilament light, pNFH, and albumin concentrations were determined in CSF samples that were collected into a polypropylene tube (Sarstedt) and in S samples that were collected into a Serum Gel with Clotting Activator tube (Sarstedt). S and CSF samples were drawn on the same day. The CSF samples were centrifuged at 390 × *g* for 10 minutes at room temperature, and the S samples were centrifuged at 2500 × *g* for 6 minutes at 4°C. Both the CSF and S samples were aliquoted into at least three vials (0.3 mL per vial) and stored at −70°C until the analysis.

### 2.3 Analytical methods

The concentrations of NFL (NF‐light^®^ [Neurofilament light] ELISA, REF 10‐7001, IVD CE, UmanDiagnostics AB) and pNFH (Neurofilament [pNf‐H] ELISA, REF EQ6561‐9601, IVD CE, Euroimmun AG; Neurofilament (pNf‐H)‐high sensitive ELISA, REF EQ6562‐9601, For Research Use Only, Euroimmun AG) were determined by ELISA. A patient sample was used for precise and reproducible measurement of NFL, as the diagnostic kit did not include a quality control sample; for measuring pNFH, the manufacturer of the diagnostic kit supplied two quality control samples. The kit manufacturers stated that the analytical sensitivity for NFL was 32 ng·L^−1^, 27 ng·L^−1^ for pNFH and 6 ng·L^−1^ for pNFH sensitive (pNFHs). All samples were measured in duplicate, and the mean intra‐assay coefficients of variation for CSF NFL, CSF pNFH, and S pNFH were 1.9%, 3.3%, and 4.2%, respectively.

### 2.4 Statistical methods

Excel, Stata version 13, MedCal version 17.9.7., and NCSS 2007 were used for the statistical analyses.^16^, ^17^ Basic descriptive statistics were used to describe the data, including frequency tables, medians, arithmetic means, standard deviations, and percentiles. The normality of the CSF NFL, CSF pNFH, and S pNFH parameters was verified with the Shapiro‐Wilk test of normality. The normality hypothesis was rejected; therefore, nonparametric tests were used, including the Kruskal‐Wallis rank test and the two‐sample Wilcoxon rank‐sum (Mann‐Whitney) test. The relationship between the parameters was evaluated by Spearman's correlation coefficient. Data values were categorized as positive and negative. Fisher's exact test was used to test categorized data. Conformity between assay results was evaluated by the kappa index with 95% confidence intervals. Statistical tests were evaluated using a 5% significance level.

### 2.5 Ethics approval

Informed consent was obtained from all patients at the University Hospital Ostrava who were included in the study. The study was approved by the Ethics Committee of the University Hospital Ostrava as a part of the project “CSF biomarkers of multiple sclerosis” (reference number 400/2017).

## 3 RESULTS

First, we partially verified diagnostic kits for NFL and pNFH determination. When we evaluated whether the measurements were precise and reproducible, both diagnostic kits showed variation coefficients that were comparable to the values supplied by the manufacturer (Table 1).

**Table 1:** Assessment of the precision and accuracy of assay methods that used either patient cerebrospinal fluid as a control for NFL or commercial controls for pNFH

Methods	Mean	SD	CV (%) (95% CI)	CV_d_ a (%)	Bias (%)
NFL (ng·L^−1^)	305.3	19.25	6.31 (4.26‐9.32)	<9.0	—
pNFH–level 1 (ng·L^−1^)	273.9	23.03	8.41 (5.91‐12.16)	6.6	−8.69
pNFH–level 2 (ng·L^−1^)	1051.1	70.03	6.66 (5.36‐8.31)	4.4	16.79

a The declared value from the manufacturer.

A total of 172 patient samples were included in the analysis that evaluated the correlation between NF levels and clinical diagnoses. The analytical characteristics of the studied group are presented in Table 2.

**Table 2:** Descriptive characteristics of the studied group

Variables	N	Median	Mean	SD	Min	Max
Age	172	46.0	47.0	16.5	12.0	85
CSF NFL (ng·L^−1^)	107	553.0	1604.8	3397.0	133.0	27 149
CSF pNFH (ng·L^−1^)	148	286.8	1099.1	3127.4	80.5	23 100
S pNFH (ng·L^−1^)	79	47.2	115.9	343.7	18.2	2916,6
S pNFHs (ng·L^−1^)	69	29.0	124.7	382.3	0.937	2705.4

**Figure 1:**
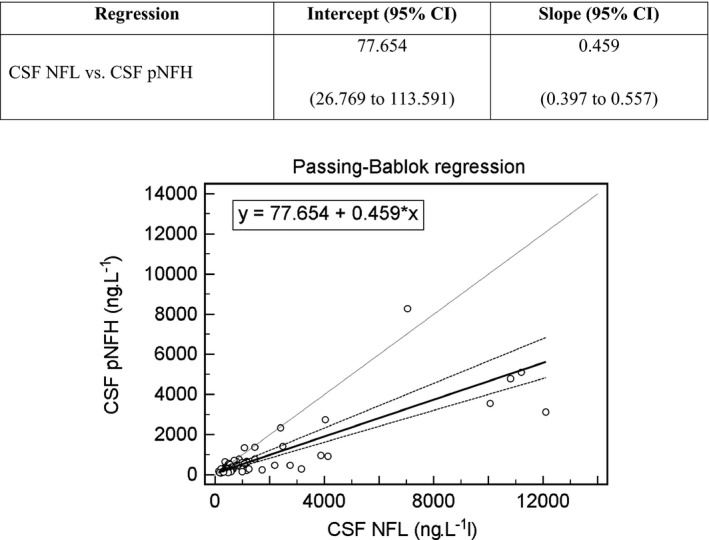
Passing‐Bablok regression analysis of CSF NFL and CSF pNFH concentrations. *r*_s_ = Spearman correlation coefficient

There was a statistically significant correlation between CSF NFL and CSF pNFH concentrations (*r*_s_ = 0.748; n = 89; *P* < 0.001). The regression relationship between these parameters was evaluated using Passing‐Bablok regression (Figure 1). At the same time**,** Passing‐Bablok regression demonstrated a statistically significant bias between the CSF NFL concentration and the CSF pNFH concentration. For a concentration of 300 ng·L^−1^, bias represents 28.11%; for a concentration of 5000 ng·L^−1^, bias is more than 50% (namely, 52.45%). This indicated that at high CSF NFL concentrations, the CSF pNFH concentration was approximately half of the CSF NFL value.

**Figure 2:**
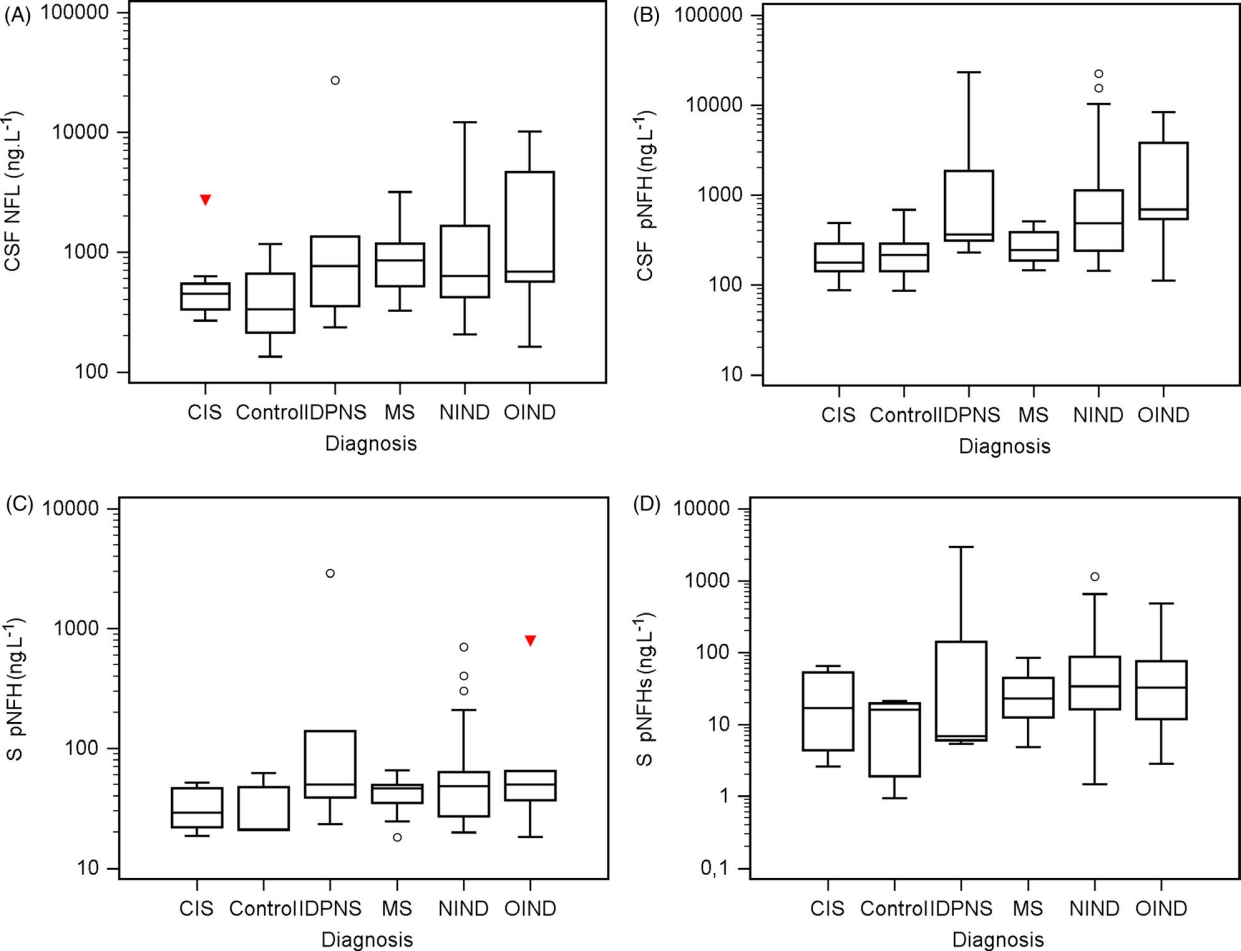
Concentrations of individual parameters according to the diagnosis. A, CSF NFL vs diagnosis; B, CSF pNHF vs diagnosis; C, S pNFH vs diagnosis; D, S pNFHs vs diagnosis

NF concentrations according to the diagnosis groups are presented in Figure 2. We evaluated the correlation between the NF concentrations and the different diagnoses. There was a statistically significant relationship between CSF NFL and CSF pNFH in the NIND (*r*_s_ = 0.793; *P* < 0.001) and control (*r*_s_ = 0.811; *P* < 0.001) diagnosis groups, and between CSF pNFH and S pNFH in the IDPNS (*r*_s_ = 0.900; *P* = 0.037) and NIND diagnosis groups (*r*_s_ = 0.459; *P* = 0.018) and between CSF pNFH and S pNFHs in the NIND diagnosis groups (*r*_s_ = 0.435; *P* = 0.030) (Table 3).

**Table 3:** Correlations between NF concentrations and the indicated diagnoses

Parameters		Diagnosis						
		MS	CIS	IDPNS	NIND	OIND	Control	All
CSF NFL vs CSF pNFH	*r*_s_ *P* n	0.396 0.144 15	0.650 0.058 9	0.400 0.600 4	0.793 <0.001 24	0.943 0.005 6	0.811 <0.001 15	0.748 <0.001 89
CSF pNFH vs S pNFH	*r*_s_ *P* n	0.368 0.177 15	0.624 0.054 10	0.900 0.037 5	0.459 0.018 26	0.657 0.156 6	0.464 0.294 7	0.579 <0.001 79
CSF pNFH vs S pNFHs	*r*_s_ *P* n	0.515 0.128 10	−0.214 0.610 8	0.800 0.200 4	0.435 0.030 25	0.300 0.624 5	0.286 0.535 7	0.439 <0.001 69

Abbreviation: *r*_s_, Spearman's correlation coefficient.

**Figure 3:**
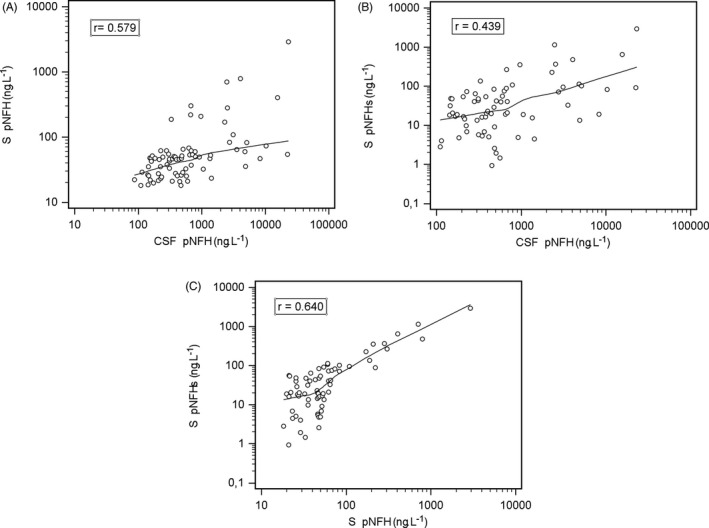
Correlation analysis of CSF pNFH and serum pNFH, CSF pNFH and S pNFHs, and S pNFH and S pNFHs

The correlation coefficient between the CSF pNFH and S pNFH values and between the CSF pNFH and S pNFHs was moderate (*r*_s_ = 0.579 resp. 0.439), probably due to the differences in the biological material that was analyzed (CSF or S; Figure 3).

The kappa statistic was used to compare the assays based on clinical interpretation because the methods had different reference intervals (Table 4). The highest kappa coefficient, that is, moderate conformity between the diagnostic kits, was demonstrated between the concentrations of CSF pNFH and NFL (*κ* = 0.556). The positive value for the concentration of CSF NFL was set at >900 ng·L^−1 ^based on the study of Arrambide et al.^15^ That is, values >900 ng·L^−1 ^can be considered to be significantly elevated and indicative of axonal damage. For CSF pNFH, the positive value was >610 ng·L^−1^ (95th percentile of negative samples; (Table 4).^18^

**Table 4:** Assay conformity based on the kappa statistic

	CSF pNFH^a^ vs CSF NFL	CSF pNFH^b^ vs CSF NFL	CSF pNFH^a^ vs S pNFH^a^	CSF pNFH^b^ vs S pNFH^b^	CSF pNFH^a^ vs S pNFHs^a^	CSF pNFH^b^ vs S pNFHs^b^
Kappa statistics Conformity (%)	0.380 (80)	0.556 (83)	0.337 (84)	0.306 (71)	0.431 (69)	0.272 (69)
95% CI	0.152‐0.608	0.360‐0.752	0.067‐0.607	0.124‐0.488	0.153‐0.709	0.088‐0.456
Standard error	0.0938	0.1043	0.0938	0.0871	0.1418	0.0940

Note

Positive values were as follows: CSF NFL > 900 ng·L^−1^ 15; CSF pNFH > 1520^a^ (respectively, 610^b^) ng·L^−1^; S pNFH > 290^1^ (resp., 130^b^), ng·L^−1^ 16.

a 95th percentile for disease controls and healthy.

b 95th percentile for negative samples.

The nonparametric Kruskal‐Wallis test was used to evaluate the relationship of individual analytes on diagnosis. A statistically significant difference was found for CSF pNFH in the group that included all diagnoses (Table 5). Post hoc analysis was performed by Dunn test (Table 6).

**Table 5:** One‐way analysis of variance of individual analytes and all diagnoses

	CSF NFL (*P*‐value)	CSF pNFH (*P*‐value)	S pNFH (*P*‐value)	S pNFH_sen. (*P*‐value)
All diagnoses^a^	0.157	<0.001	0.138	0.260

a Kruskal‐Wallis rank test.

**Table 6:** Mutual comparison of individual diagnoses (Dunn test)

Diagnosis groups (n)	Average rank	Different (*P* < 0.05) from subgroups
(1) CIS (20)	41.40	(5)
(2) Control (24)	49.64	(4) (5)
(3) IDPNS (5)	101.00	
(4) NIND (37)	82.93	(2)
(5) OIND (10)	112.88	(1) (2)
(6) MS (14)	63.53	

We also investigated the correlations of NF concentrations with clinical data. The CSF NFL concentration in the MS and CIS diagnosis groups was significantly higher in the subgroup of patients who had a expanded Kurtzke Disability Status Scale (EDSS) score of 2.5 or higher (median 1208.5 ng·L^−1^) 6 months after lumbar puncture versus the subgroup of patients with EDSS scores of 0 to 2 (median 488 ng·L^−1^; *P* = 0.0269; Table 7). CSF NFL thus appears to be a promising parameter for predicting disability severity.

**Table 7:** Correlation of the CSF NFL concentration with clinical data (MS and CS group, patients with available follow‐up data, Mann‐Whitney test)

	CSF NFL median (IQR)	
Relapse at the time of lumbar puncture		
No	883 (533‐1471)	n = 18/8 ns *P* = 0.2013
Yes	607 (439.5‐992.5)	
Relapse within 6 mo after lumbar puncture		
No	1083 (488‐1471)	n = 14/3 *P* = 0.1015
Yes	446 (N/A)	
Kurtzke Expanded Disability Status Scale (EDSS) 6 mo after lumbar puncture		
0‐2.0	488 (438.5‐1039)	n = 9/8 *P* = 0.0269
≥2.5	1208.5 (862‐1597.5)	

## 4 DISCUSSION

This study investigated NF concentrations in S and in CSF as a marker of axonal damage. We used two CE ELISA diagnostic kits, one to determine the concentrations of NFL in CSF (NF‐light ELISA, UmanDiagnostics) and one to determine the concentrations of pNFH in S and CSF (Neurofilament (pNf‐H) ELISA, Euroimmun). The other ELISA diagnostic kits for S pNFHs determination (Neurofilament (pNf‐H)‐high sensitive ELISA, Euroimmun) are for research use only. All three assays were suitable for precise measurement of NFs. There was a statistically significant correlation between CSF NFL levels and CSF pNFH levels. However, the CSF pNFH assay showed much lower values than the values for CSF NFL. The reason for this difference may be the relative molar ratio of the individual NF subunits, which is approximately 5:2:1 for NFL:NFM:NFH.^19^ Another possibility is that NFL, which has a lower molecular mass, diffuses into CSF more easily than the heavier pNFH or with regard to the NF stoichiometry as motor neurons have the ability to save energy to shift the protein expression from larger to smaller subunits.^20^

We demonstrated that the CSF NFL concentrations correlated well with the CSF pNFH concentrations, especially in the Control and in patients with NIND. We found no correlation for the other diagnosis groups, possibly due to the small number of patients, especially in the IDPNS group. The correlation between the S and CSF concentrations of pNFH was lower. The SIMOA method is a suitable alternative for testing these analytes in CSF and S. Kuhle et al^11^, ^21^, ^22^ showed that SIMOA had higher sensitivity than ELISA and the electrochemiluminescence‐based assay. They further reported a statistically significant correlation between CSF NFL and S NFL concentrations. In the future, despite the high cost of the SIMOA method, it would be appropriate to examine a larger dataset to determine whether S NFL determination could replace CSF NFL determination for assessing the severity and prognosis of neurological diseases.

When we evaluated the correlations of these methods with the different diagnoses, the assays showed the best correlations with each other in the IDPNS and NIND groups of patients. Similar results were obtained by De Schaepdryver et al,^22^ who determined the S and CSF NF concentrations using two diagnostic kits from Euroimmun and BioVendor. Both kits were ELISA‐based. The authors compared the assays in a group of patients with amyotrophic lateral sclerosis and showed a good correlation between CSF NFH and S NFH (*r* = 0.652).

When we evaluated these assays according to clinical findings, there was moderate conformity between the CSF NFL and pNFH concentrations, but only fair conformity between the CSF pNFH and the S pNFH concentrations. One possible explanation is that NFs are heteropolymers that form aggregates. Thus, precise determination of NFH concentrations by immuno‐based methods can be influenced by several factors: the ability of the aggregate to mask the NFH epitope; the aggregate's decreased solubility; the difference in stability of NFH monomers in solution versus NFH in aggregates; and the ability of the antibody to bind to soluble NFH.^23^ Lu et al^23^ confirmed that NF aggregates are characteristic of amyotrophic lateral sclerosis and other neurodegenerative diseases and that they represent a significant pre‐analytical problem for immunoassay analysis. They developed an ELISA method in which, after 1‐hour incubation of the sample with a buffer containing a “urea‐calcium chelator,” aggregate disruption resulted in a precise quantification of the NFH concentration.

We performed one‐way analysis of variance of the individual analytes for all diagnoses and for paired comparisons of patients with MS and CIS versus the control group. A statistically significant difference was found only for the concentration of CSF pNFH in the group that included all diagnoses. This finding was not a surprise, because a number of studies have shown that NFs are markers of axonal damage rather than markers of a specific diagnosis. Increased levels of NF have been observed, for example, in ALS,^22^ CIS/MS,^4^, ^5^, ^6^, ^7^, ^8^, ^9^, ^10^, ^12^, ^13^, ^14^, ^15^ neurological diseases related to aquaporin‐4‐ (AQP4‐Ab‐) and myelin oligodendrocyte glycoprotein antibodies (MOG‐Ab), and other neurological diseases.^24^

We studied the correlation of CSF NFL with clinical data as well. The CSF NFL concentration in the MS and CIS diagnosis groups was significantly higher in the subgroup of patients who had EDSS scores of 2.5 or higher 6 months after sampling versus the subgroup of patients with EDSS scores of 0 to 2. These data showed the suitability of using CSF NFL to predict disease severity. Similar results were obtained in the study by Disanto et al,^25^ which examined the relationship between NFL concentration and other markers of disease activity, such as the number of T2 hyperintense and gadolinium‐enhancing (Gd+) lesions on cranial MRI and the presence of IgG oligoclonal bands in the CSF of patients with CIS. That group found higher S NFL concentrations in patients with T2 and Gd+ lesions, and the NFL concentrations increased with increasing EDSS scores at CIS time. These results are in line with other studies showing that NF levels in CSF are correlated with both MRI and clinical markers of MS disease activity.^26^

## 5 CONCLUSION

In this study, we tested three diagnostic kits for the determination of NF concentrations in biological fluids. The NFL ELISA assay had lower sensitivity and was suitable only for CSF analysis, while the pNFH ELISA assay had satisfactory sensitivity and was suitable for S and CSF analysis, the pNFHs only for S analysis. The data showed good correlation and moderate conformity between CSF NFL and CSF pNFH concentrations, indicating that the results can be considered to be consistent. However, the low correlation coefficient and the kappa index found between the S pNFH, even if using a high‐sensitivity ELISA assay and CSF pNFH meant that the S pNFH and S pNFHs assays gave a lower predictive value. When assessing the relationship of NF concentrations and diagnosis, correlations were found between the concentration of CSF NFL and CSF pNFH in the NIND diagnosis group and in the control group of patients, between the CSF and S pNFH in the IDPNS and NIND diagnosis groups, and between the CSF and S pNFHs in the NIND diagnosis groups. The results confirmed that NFs, whether NFLs or pNFHs, represent an etiologically nonspecific indicator of tissue damage and that it is better to determine their levels in CSF than in S.

## CONFLICT OF INTEREST

The authors report no conflicts of interest. The authors alone are responsible for the content and writing of the paper.
